# Covalent Adsorption of N-Heterocyclic Carbenes on a Copper Oxide Surface

**DOI:** 10.1021/jacs.2c06335

**Published:** 2022-09-01

**Authors:** Juan J. Navarro, Mowpriya Das, Sergio Tosoni, Felix Landwehr, Jared P. Bruce, Markus Heyde, Gianfranco Pacchioni, Frank Glorius, Beatriz Roldan Cuenya

**Affiliations:** † Department of Interface Science, Fritz-Haber Institute of the Max-Planck Society, 14195 Berlin, Germany; ‡ Westfälische Wilhelms-Universität Münster, Organisch-Chemisches Institut, 48149 Münster, Germany; § Dipartimento di Scienza dei Materiali, Università di Milano-Bicocca, Via Cozzi 55, 20125 Milano, Italy

## Abstract

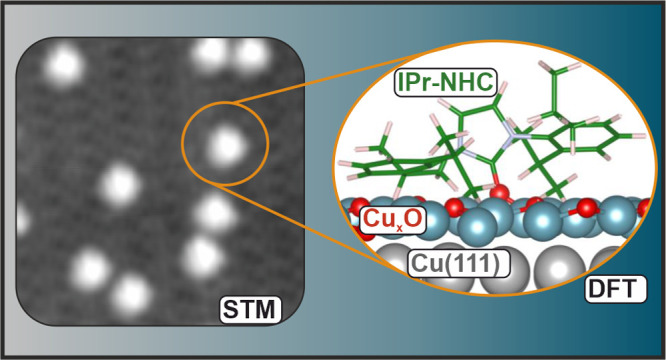

Tuning the properties of oxide surfaces through the adsorption of designed ligands is highly desirable for several applications, such as catalysis. N-Heterocyclic carbenes (NHCs) have been successfully employed as ligands for the modification of metallic surfaces. On the other hand, their potential as modifiers of ubiquitous oxide surfaces still needs to be developed. Here we show that a model NHC binds covalently to a copper oxide surface under UHV conditions. In particular, we report the first example of a covalent bond between NHCs and oxygen atoms from the oxide layer. This study demonstrates that NHC can also act as a strong anchor on oxide surfaces.

The functionalization of oxide surfaces through the covalent attachment of molecular monolayers has been intensively pursued,^1^ leading to very important advances in the fields of optoelectronics, biosensing, and catalysis.^2−4^ Different approaches were employed to achieve this goal, including the use of silanes, phosphonates, carboxylates, and thiols.^1,5,6^ N-Heterocyclic carbenes (NHCs) have been successfully employed in the modification of metal surfaces due to their capability of forming strong bonds to metallic centers.^7−14^ Furthermore, it is possible to tune the binding mode by carefully selecting the side groups.^15^ Less common is the attachment of NHC on semiconductors,^16^ and the direct binding of NHCs to metal oxides was not reported to date.^17−19^ In particular, mainly transition-metal NHC complexes were employed to functionalize metal oxide particles.^20−25^

Many metal surfaces present a native oxide under ambient conditions, which can also participate in the adsorption of ligands. Among these metals, copper, an abundant and inexpensive first-row transition metal,^26^ is historically one of the most commonly employed in the development of technological applications. The functionalization of oxidized copper surfaces is challenging because the attachment of organic molecules leads to reduction.^27−30^ At the same time, many efforts have been made to avoid further oxidation of copper using thiols or, recently, NHC ligands.^31−33^ In photocatalysis, copper oxide is a widely used material and the attachment of organic molecules can be very beneficial.^34^

In this work, we study the adsorption of a model NHC (1,3-bis(2,6-diisopropylphenyl)imidazol-2-ylidene, IPr-NHC) molecule on a copper oxide layer grown on Cu(111) by means of low-temperature scanning tunneling microscopy (LT-STM), X-ray photoelectron spectroscopy (XPS), and density functional theory (DFT). We show that the IPr-NHC molecules strongly bind to the surface without distorting the long-range order of the oxide layer. Furthermore, we demonstrate that IPr-NHC forms a covalent bond with the oxygen atoms from the oxide layer, representing the first example of NHC attachment on a metal oxide where no metal complex is needed.

IPr-NHC molecules adsorb on the bare Cu(111) surface, forming a hexagonal lattice and well-defined structures such as the molecular islands in Figure 1a.^35^ This image corresponds to 0.25 ML of IPr-NHC on Cu(111). Occasionally, some molecules move during scanning (for example, the ones marked in Figure 1a), indicating a certain mobility under specific tunneling conditions. The formation of an oxide layer (Cu_*x*_O) on Cu(111), as described in the Supporting Information (SI), results in a variety of structures depending on the amount of oxygen incorporated. In this case, most of the surface is covered by the “29” structure,^36−38^ with some patches of the “41” structure.^39^ Both phases exhibit a characteristic row pattern. The evaporation of 0.05 ML (according to the calibration on bare Cu(111)) of IPr-NHC on the Cu_*x*_O layer results in the arrangement shown in Figure 1b,c for the 29 and 41 phases, respectively. The observed arrangement on Cu_*x*_O contrasts dramatically with the one on the bare Cu(111) surface. In particular, two properties for the arrangement on Cu_*x*_O are worth mentioning: (1) The ligands do not form close-packed structures. (2) No molecular mobility is observed for a broad range of bias voltages (section C in the SI). Regarding the adsorption on the different oxide phases, the 41 regions present a higher coverage in comparison to the 29 regions, suggesting a certain difference in reactivity.

**Figure 1:**
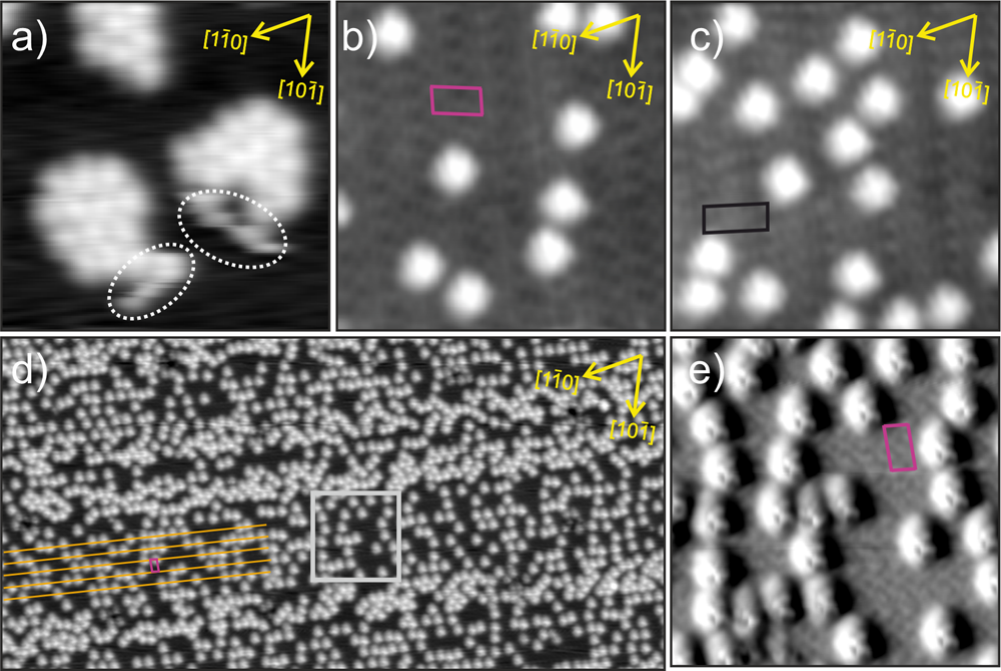
IPr-NHC molecules on (a) Cu(111) and (b–e) Cu_*x*_O. (a) On Cu(111): 13 nm × 13 nm, *V*_s_ = 1.5 V, and *I*_t_ = 20 pA. The motion of molecules is marked (white circles). (b) On the “29” Cu_*x*_O structure: 13 nm × 13 nm, *V*_s_ = −1.0 V, and *I*_t_ = 20 pA. (c) On the “41” Cu_*x*_O structure: 13 nm × 13 nm, *V*_s_ = −1.0 V, and *I*_t_ = 20 pA. (d) Higher coverage on a larger area of the Cu_*x*_O surface: 100 nm × 50 nm, *V*_s_ = 1.0 V, and *I*_t_ = 80 pA. (e) Derived from the area marked in gray in panel (d): 13 nm × 13 nm. Pink and black rectangles mark the “29” and “41” Cu_*x*_O unit cells, respectively. Orange lines indicate the direction of the stripes formed by the “29” Cu_*x*_O structure. The Cu(111) high-symmetry directions are marked by yellow arrows.

Figure 1d shows the Cu_*x*_O surface after depositing 0.25 ML of IPr-NHC (coverage according to the calibration on the bare Cu(111) surface). A stripe pattern can be clearly recognized. The molecules arrange, forming rows especially in the regions with a lower density of molecules (orange lines). Interestingly, the distance between these rows matches the long lattice vector of the 29-Cu_*x*_O structure. The magnification shown in Figure 1e shows how the molecules are actually confined in the row pattern from the 29-Cu_*x*_O lattice, meaning that the molecular arrangement is strongly influenced by the substrate. In addition, the oxide structure is not distorted by the increased molecular coverage. The regions of the stripe pattern showing a higher density of molecules and poor order are, because of the relative quantity, probably related to the 41-Cu_*x*_O areas, indicating a lower site selectivity inside its unit cell.

The adsorption of IPr-NHC on Cu_*x*_O has been modeled by means of static structural relaxation with dispersion-corrected DFT. The complex potential energy surface was partially explored by studying three possible adsorption modes: chemisorption with the formation of a carbene–oxygen bond (NHC–O, Figure 2a,b), chemisorption with the formation of a carbene–Cu bond (NHC–Cu, Figure 2c,d), and aspecific physisorption (Figure 2e,f). The results are collected in Table 1. In NHC–O, the ligand binds to the support with a very large adsorption energy, *D*_e_ = −5.01 eV, and a C–O bond distance of 1.26 Å. The ligand is able to break a Cu–O bond in the oxidized overlayer, and the oxygen bound to the carbene center points outward from the surface. This structure may be a stable intermediate toward the reduction of the oxidized copper substrate by means of organic ligands. A less favorable though strongly bound configuration is obtained if IPr-NHC binds to a Cu atom from the Cu_*x*_O overlayer (*D*_e_ = −3.85 eV). Also in this case, a Cu–O bond is broken and the Cu atom is dragged out from the surface to bind the ligand (the C–Cu distance is 1.85 Å). It is interesting to compare these results with those obtained at the same level of calculations on the clean Cu(111) and Cu(100) surfaces, where IPr-NHC was found to attach to the surface with adsorption energies of as large as 3.7–4.20 eV while still being able to diffuse on the surface-forming islands and assemblies.^35^ The remarkably larger *D*_e_ reported for the most stable structure, NHC–O, is a first hint explaining the nonmobile behavior of IPr-NHC on oxidized supports. A second, important aspect is that on Cu(111) the stable adsorption sites for the ligand are very close to each other, while in the present case a diffusion via desorption/readsorption necessarily implies the breaking of a strong C–O covalent bond. The least-stable configuration is the one envisaging only nonspecific dispersive interactions between the ligand and the surface, exerted by the large isopropylphenyl side substituents. This corresponds to a local minimum with *D*_e_ = −1.96 eV.

**Figure 2:**
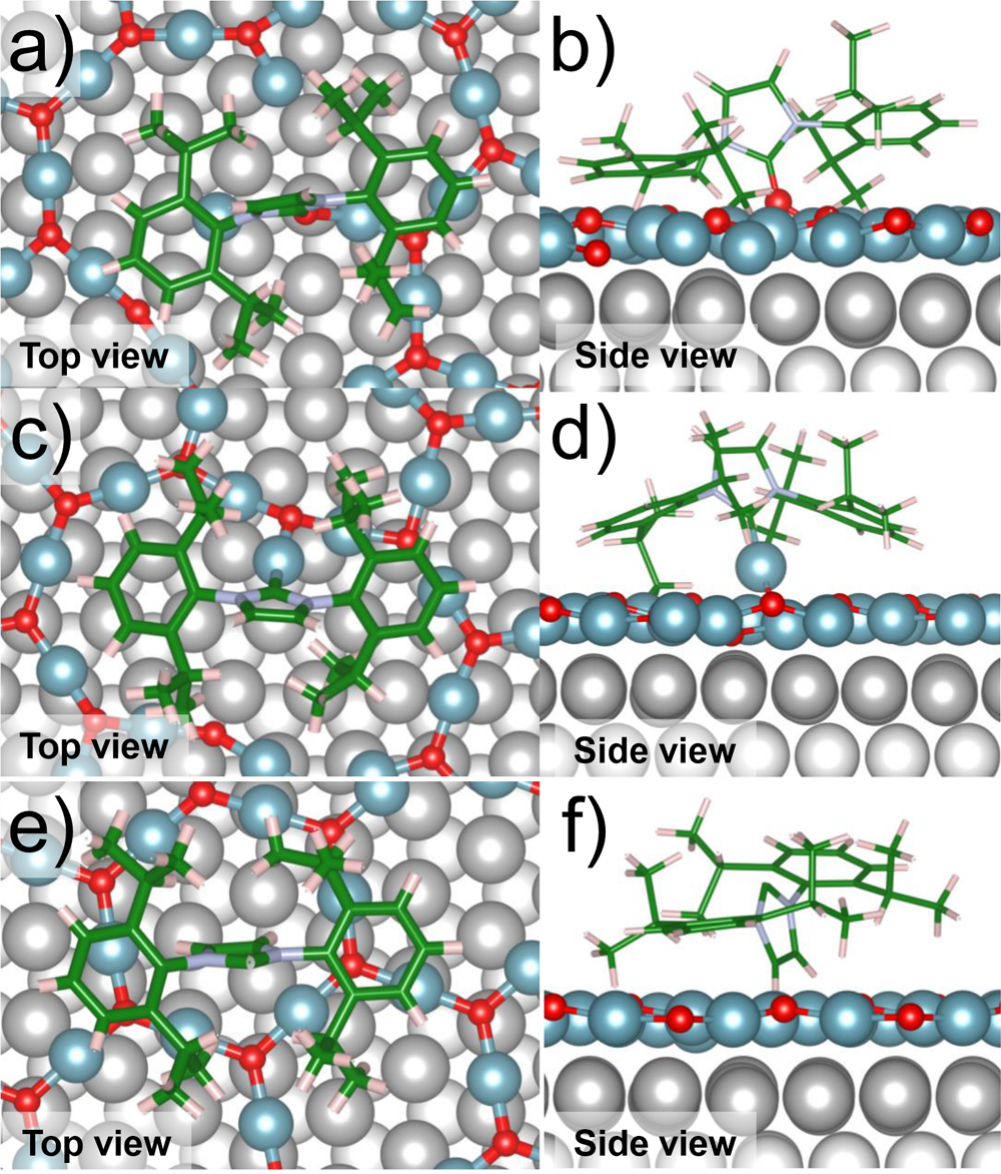
DFT calculations of IPr-NHC on Cu_*x*_O. (a) Top and (b) side views of the NHC–O bond configuration. (c) Top and (d) side views of the NHC–Cu bond configuration. (e) Top and (f) side views of physisorbed IPr-NHC. Cu from Cu(111) (greys), Cu from Cu_*x*_O layer (metallic blue), O (red), C (green), N (light violet), and H (light pink).

**Table 1:** Calculated Adsorption Energies and Bond Lengths of IPr-NHC on Different Supports and Sites by Means of DFT

support	bond	*D*_e_ (eV)	bond length (Å)
Cu(111)	NHC–Cu	–3.68	1.98
Cu_*x*_O	NHC–Cu	–3.85	1.85
Cu_*x*_O	NHC–O	–5.01	1.26
Cu_*x*_O	physisorption	–1.96	

The role of the side substituents in terms of the additional stabilization of IPr-NHC is sizable in NHC–O and NHC–Cu as well, where the long-range dispersion accounts for 65 and 51% of *D*_e_. If phenyl (or smaller) groups are adopted instead of diisopropylphenyl, then NHC–O and NHC–Cu binding modes display the same stability (section G in the S.I.), highlighting the role of steric hindrance in determining the binding mode. Previous studies showed the strong influence of the side substituents in the binding mode of NHCs on metallic surfaces.^15^ While the diisopropylphenyl groups used in the present study lead to vertical adsorption,^10,35^ other side substituents favor a lying configuration, lifting a metallic atom from the substrate and forming mononuclear complexes.^11,40,41^ On polycrystalline copper oxide, a treatment with 1,3-diisopropylbenzimidazoliumhydrogen carbonate results in the formation of a cyclic urea and an NHC copper complex.^30^

The formation of a covalent bond between IPr-NHC and the O atoms from the Cu_*x*_O is further supported by XPS measurements. Figure 3a,b show the O 1s spectra for the as-prepared Cu_*x*_O and for the IPr-NHC adsorbed on Cu_*x*_O, respectively. For the as-prepared Cu_*x*_O surface, the observed O 1s peak appears at 529.5 eV (Figure 3a), in agreement with previous studies.^43−46^ After the deposition of IPr-NHC, a new component at higher binding energy, 531.3 eV, appears (Figure 3b). In addition, the original peak found in the as-prepared Cu_*x*_O sample is now located at 529.7 eV. Our DFT calculations predict a shift of +0.6 eV toward higher binding energies for the O 1s core level of those oxygen atoms that, forming part of the Cu_*x*_O lattice, bind to an IPr-NHC molecule. This shift can be related to the new component appearing in Figure 3b. The underestimation of the calculated shift with respect to the XPS data (where the new O 1s component is shifted +1.6 eV with respect to the original one) may depend on several factors, such as the neglection of final state effects and the overestimation of the electron delocalization in the proximity of a metal substrate, common to DFT. This may affect the screening of the surrounding Cu 3d states on the O 1s core levels.

**Figure 3:**
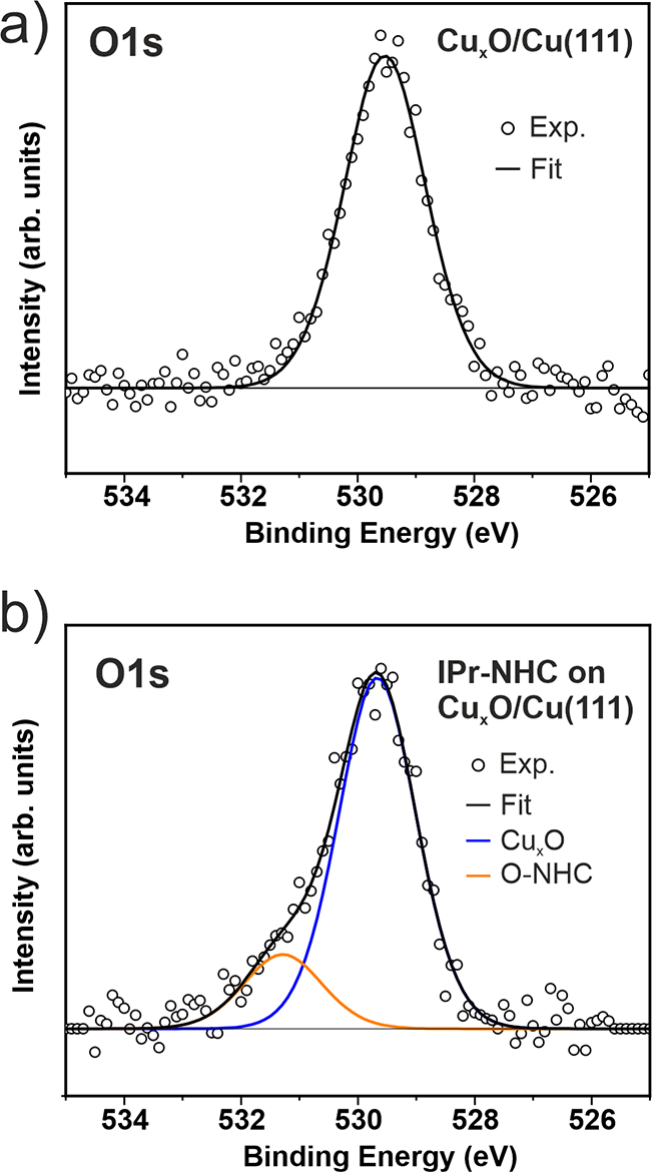
O 1s spectra recorded on (a) Cu_*x*_O/Cu(111) and (b) 1 ML IPr-NHC on Cu_*x*_O/Cu(111). A Shirley background has been subtracted.^42^

The qualitative agreement between DFT and XPS data supports the idea of IPr-NHC ligands forming a covalent bond to oxygen atoms from the oxide layer. The formation of bonds between NHCs and oxygen atoms is well reported for the synthesis of cyclic ureas.^47−51^ In the present work, however, the binding oxygen atom preserves the bond with the oxide layer (Figure 2a,b). The binding oxygen atom thus acts as an anchor atom (section G in the S.I.), fixing the IPr-NHC molecule on the Cu_*x*_O layer. This strong attachment provides good thermal stability of the ligands, even at temperatures of up to 420 K (section H in the S.I.). Interestingly, the functionalization of oxide surfaces takes place normally with the NHC group forming a metal complex.^18−25^ In the present study, the carbene centers can bind directly to the O atoms from the Cu_*x*_O layer.

To conclude, IPr-NHC successfully attaches on a Cu_*x*_O layer grown on Cu(111). A strong interaction between the ligands and the substrate is supported by STM measurements, revealing a molecular arrangement governed by the Cu_*x*_O structure. DFT calculations found that the most stable molecular configuration for IPr-NHC on Cu_*x*_O/Cu(111) is one in which IPr-NHC binds covalently to an O atom from the Cu_*x*_O layer, predicting a shift of the O 1s level toward higher binding energies. The XPS data corroborate this energy shift. Our study demonstrates that NHCs anchor strongly to the Cu_*x*_O lattice through oxygen atoms from the oxidized surface, exhibiting thermal stability at temperatures of up to 420 K. NHC ligands thus present a promising way to tune the properties of oxide surfaces in a wide range of applications even without employing metal complexes.

## Supporting Information Available

Methods; copper oxide structures on Cu(111); STM of IPr-NHC on Cu_*x*_O at different bias voltages; apparent size of IPr-NHC from STM images; Cu 2p, CuLMM, C 1s, and N 1s XPS peaks; DFT calculations for different substituents; and thermal stability of IPr-NHC on Cu_*x*_O/Cu(111) (PDF)
