# Supplementary material for: Covalent Adsorption of N-Heterocyclic Carbenes on a Copper Oxide Surface
Source: J Am Chem Soc. 2022 Sep 1;144(36):16267–71. doi: 10.1021/jacs.2c06335 (PMC9479068; doi:10.1021/jacs.2c06335)
Supplement: Supplementary file 1 — ja2c06335_si_001.pdf [file ja2c06335_si_001.pdf]

Supporting information

Covalent Adsorption of N-Heterocyclic Carbenes on a Copper Oxide Surface

Juan J. Navarro,[†] Mowpriya Das,[‡] Sergio Tosoni,[*,¶] Felix Landwehr,[†] Jared P. Bruce,[†] Markus Heyde,[*,†] Gianfranco Pacchioni,[¶] Frank Glorius,[*,‡] and Beatriz Roldan Cuenya[†]

† Department of Interface Science, Fritz-Haber Institute of the Max-Planck Society, 14195 Berlin, Germany

‡ Westfälische Wilhelms-Universität Münster, Organisch-Chemisches Institut, 48149 Münster, Germany

¶ Dipartimento di Scienza dei Materiali, Università di Milano-Bicocca, Via Cozzi 55, 20125 Milano, Italy

Corresponding authors:

[*] ST, sergio.tosoni@unimib.it; MH, heyde@fhi-berlin.mpg.de;
FG, glorius@uni-muenster.de

INDEX

A. Methods ……………………………………………………………………........S2

B. Copper oxide structures on Cu(111) ……………………………………......S3

C. STM Images at different bias from IPr-NHC on Cu_xO/Cu(111) ..……...S4

D. Apparent size of IPr-NHC on Cu(111) and Cu_xO/Cu(111)……………...S5

E. XPS: Cu_xO/Cu(111) before and after deposition of IPr-NHC………… S6

F. XPS: N1s peak. IPr-NHC on Cu_xO/Cu(111) and Cu(111)………………..S7

G. Binding modes and effect of the N-substituents ……………………….S8

H. Thermal Stability of IPr-NHC on Cu_xO/Cu(111)…………………………....S10

REFERENCES S12

A. Methods

The synthesis of 1,3-bis(2,6-diisopropylphenyl)-1*H*-imidazol-3-ium-2-carboxylate (IPr-CO_2 adduct) was carried out as described in previous publications.[1] The ligands were deposited on the substrate under UHV conditions (~ 5 $\times 10^{-10}$ mbar). The IPr-CO_2 adduct was heated in a Knudsen cell at 340 K, resulting in the generation of the free IPr-NHC with only CO_2 as a by-product. The deposition rate of IPr-NHC on the bare Cu(111) with the sample at 310 K is 0.05 monolayers/min. The same evaporation conditions were employed for the deposition on Cu_xO/Cu(111).

Cycles of sputtering with Ar^+ at 1 kV for 30 minutes and annealing at 950 K for 5 minutes were employed to prepare a clean Cu(111) surface. Afterwards, the surface was exposed to a 5 × 10^{-7} mbar partial pressure of molecular oxygen while annealing at 670 K for 30 min. Finally, the surface was annealed at 720 K in UHV for 5 min, resulting in a copper oxide monolayer as the one presented in Section B of the Supporting Information. IPr-NHC molecules were evaporated on the Cu_xO surface held at 310 K, leading to the arrangements shown in Figure 1 of the main text. The observed structures do not change after annealing at temperatures as high as 420 K (see section H), in agreement with the high stability of the IPr-NHC ligands on the Cu_xO surface discussed in the main text.

The STM images were taken at 5 K under pressures < 2 $\times 10^{-10}$ mbar using a PtIr tip, in constant current mode. In the main text, V_s denotes the bias voltage at the sample and I_t the tunneling current. XPS measurements were performed employing a X-ray source with an Al anode operating at 200 W and a Phoibos100 analyzer. The background of the spectra was substracted through the Shirley method. For the fittings, Gaussian-Lorentzian functions were employed.

All calculations were done with the code VASP 6.[2] The core electrons were modelled with the Projector Augmented Wave (PAW) method,[3-4] while H(1s), C(2s,2p), N(2s,2p), O(2s,2p) and Cu(3d,4s) electrons were treated explicitly with a set of plane waves expanded up to a kinetic energy cutoff of 400 eV. The PBE exchange-correlation functional[5] was adopted, including the long-range dispersion according to the DFT+D2' scheme.[6-7] Structure relaxations were performed with thresholds of 10^{-5} eV and 10^{-2} eV/Å for electronic and ionic loops, respectively. The sampling in the reciprocal space was reduced to the Γ point due to the large dimension of the supercells. The dipole correction was applied along the non-periodic direction and an empty layer of at least 15 Å thickness was included in the supercell. The Cu lattice constant was at first relaxed, yielding 3.57 Å. The Cu(111) surface was then simulated by a five-layers slab, where the ionic coordinates of the three top-most layers were relaxed, while the ions from the two bottom layers were frozen in their bulk positions. A cuprite (111) single layer was then added on top of a 7×7 Cu(111) supercell (17.67 Å × 17.67 Å) with a rotation of 11° with respect to the underlying Cu(111) support.[8] Adsorption of a single Ipr-NHC carbene molecule was then simulated on this supercell, allowing relaxation of the molecule as well as the substrate (beside the two bottom Cu layers). The adsorption energy, D_e, is defined as the energy of the molecule/substrate adduct with respect to the energy of its separated components:

$$D_e = E(IPrNHC/Cu_xO/Cu) - [E(IPrNHC)+E(Cu_xO/Cu)] \quad (1)$$

Negative values of D_e imply stable bonding.

B. Copper oxide structures on Cu(111)

Depending on the amount of oxygen incorporated in the layer, different structures can be observed in Cu_xO/Cu(111). Ordered structures require annealing of the surface, which can lead to a Cu_2O(111)-like surface[9] or, as more oxygen is incorporated, to the “44” and “29” phases.[10-12] In the present work, the thermal oxidation of the Cu(111) surface under the conditions specified in the Methods section leads to the structures observed in Figure S1a. Most of the surface is covered by the “29” structure, whose unit cell is indicated in Figure S1b. Some stripes from an additional phase can also be identified. The corresponding unit cell is shown in Figure S1c, and matches with the recently reported “41” structure.[13] This phase is similar to the “44” structure but with a slightly higher oxygen content.

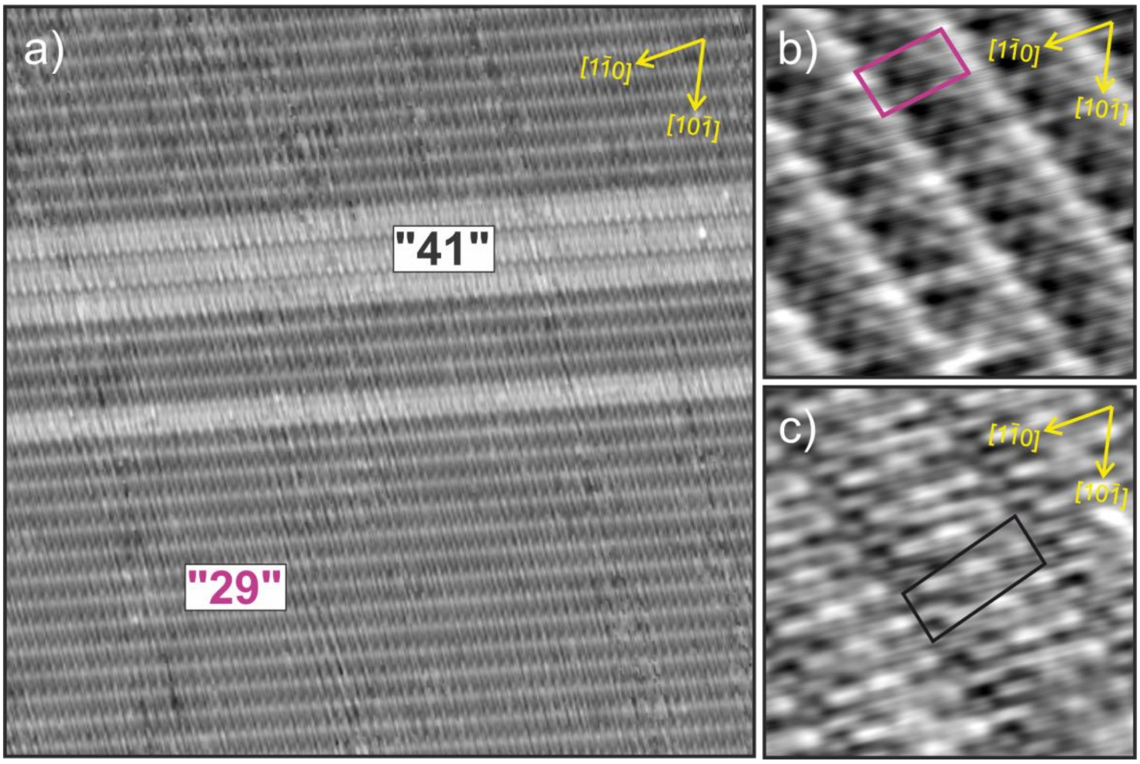

Figure S1. Copper oxide layer on Cu(111). a) 57 nm × 57 nm, V_s = -1.3 V, I_t = 50 pA. High symmetry directions of Cu(111) marked by yellow arrows. b)”29” Structure. 7 nm × 7 nm, V_s = +1.0 V, I_t = 50 pA. c) “41” Structure. 7 nm × 7 nm, V_s = +0.8 V, I_t = 50 pA.

C. STM Images at different bias from IPr-NHC on Cu_xO/Cu(111)

A way of evaluating the stability of the ligands on the surface is the adquisition of STM images at different bias voltages. As can be seen in Figure S2, no mobility is observed at bias voltages as high as +3.0 V. It is important to note that the STM images are recorded at 5 K and no mobility due to thermal diffusion is expected. Therefore, the interactions tip-molecule and substrate-molecule are the decisive factors here. Since the same molecule on Cu(111) presents a certain degree of mobility under scanning conditions (Figure 1a), the STM images from Figure S2 suggest a stronger molecule-substrate interaction on the oxidized substrate.

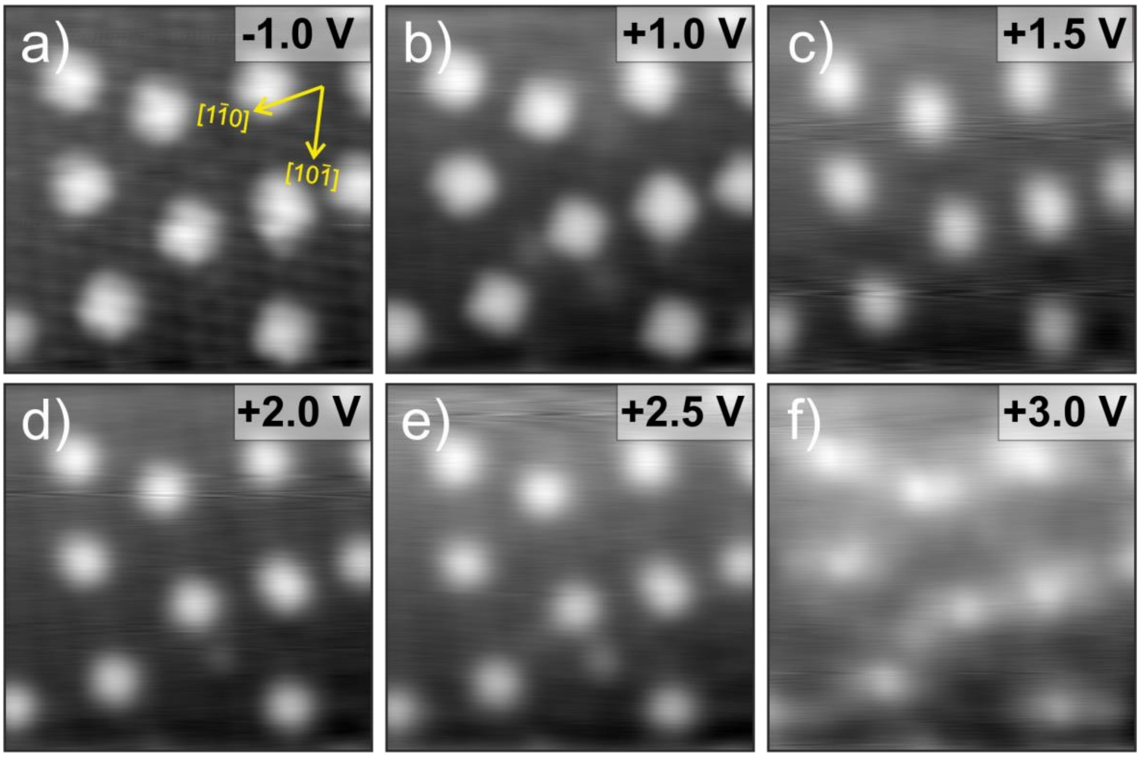

Figure S2. STM bias series of IPr-NHC on Cu_xO/Cu(111). 10 x 10 nm, I_t = 20 pA, a) V_s = -1.0 V, b) V_s = +1.0 V, c) V_s = +1.5 V, d) V_s = +2.0 V, e) V_s = +2.5 V, f) V_s = +3.0 V. High symmetry directions of Cu(111) marked by yellow arrows in panel a).

D. Apparent size of IPr-NHC on Cu(111) and Cu_xO/Cu(111)

To compare the apparent size of the molecules, we select STM images taken at the same bias voltage, like those shown in Figure S3a-b. On Cu(111) most of the molecules form islands, but occasionally it is possible to find an individual molecule as observed in Figure S3a. Individual molecules on Cu(111) appear in the STM images slightly broader than those forming islands. On Cu_xO, because of the stronger molecule-substrate interaction, the molecules adsorb individually. The width of the molecules on Cu_xO is slightly broader (~ 4% for the selected molecules in Figure S3c) than on Cu(111). Since the IPr-NHC molecules adsorb in upright configuration on Cu(111), we have assumed a similar configuration on Cu_xO, in agreement with DFT calculations (Figure 2). In addition, no node is observed in the height profiles, so the formation of dimers can be excluded.

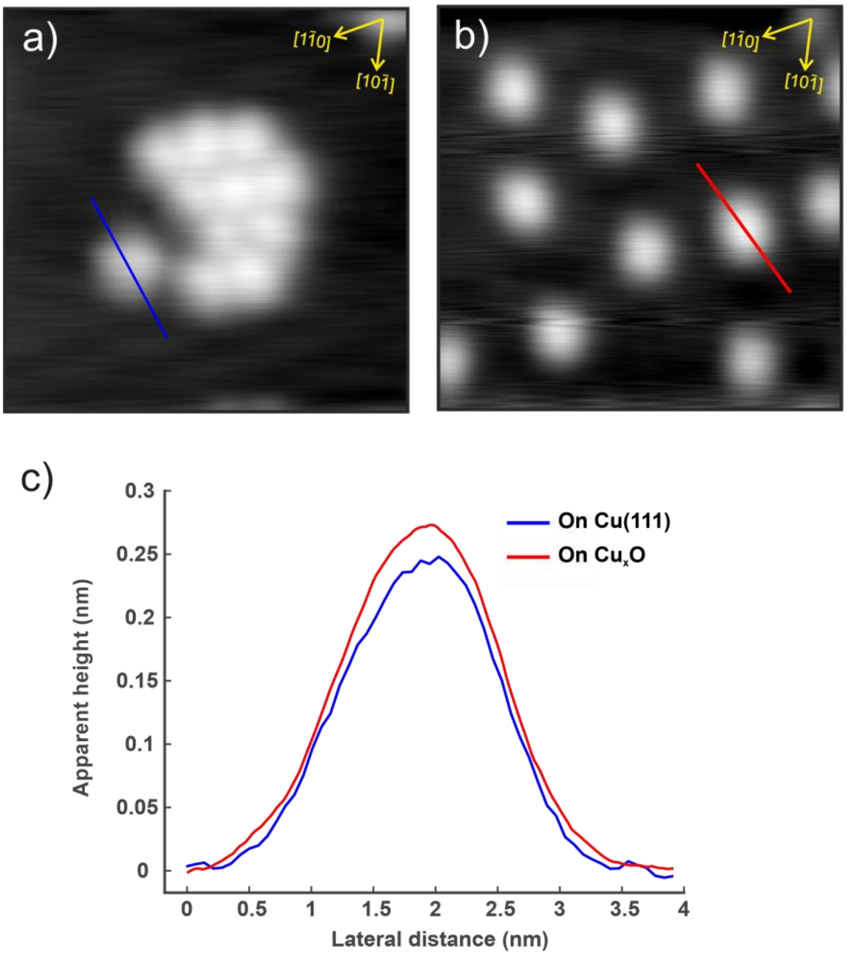

Figure S3. Apparent size of IPr-NHC molecules on Cu(111) and on Cu_xO. a) STM image showing IPr-NHC molecules on Cu(111). 10 nm x 10 nm, V_s = 1.5 V, I_t = 20 pA. b) STM image showing IPr-NHC molecules on Cu_xO. 10 nm x 10 nm, V_s = 1.5 V, I_t = 50 pA. Adapted from Figure S2c. High symmetry directions of Cu(111) are marked by yellow arrows. c) Height profiles taken along single molecules on Cu(111) (blue) and Cu_xO (red).

E. XPS: Cu_xO/Cu(111) before and after deposition of IPr-NHC

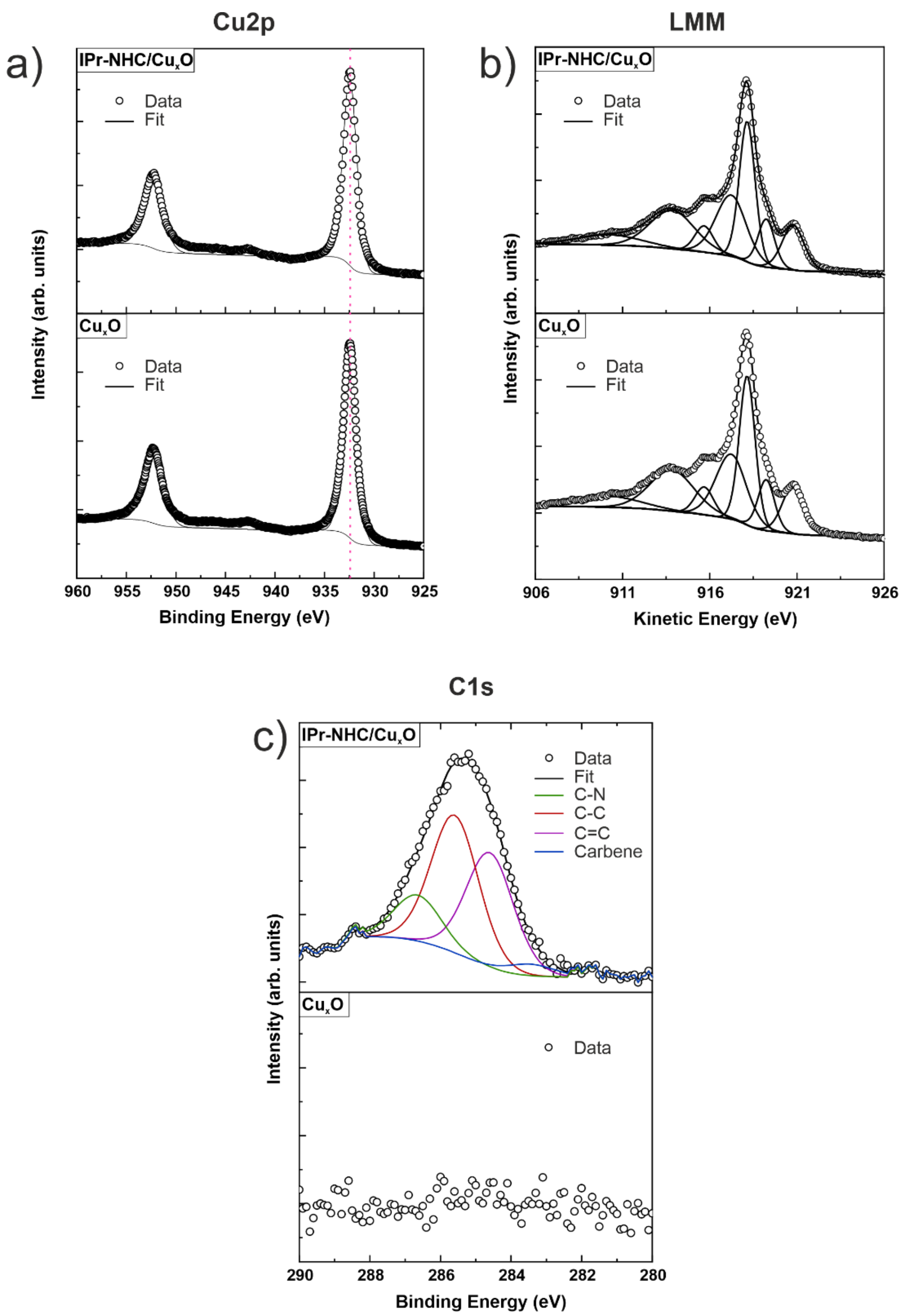

Figure S4. XPS data taken before and after the deposition of IPr-NHC on Cu_xO/Cu(111). a) Cu2p, red dotted line indicating the $Cu2p_{3/2}$ peak at 932.6 eV. b) Cu LMM Auger spectra. c) C1s spectra. In the top panel, the different components correspond to C-N (green, 286.6 eV), C-C (red, 285.6 eV), C=C (pink, 284.6 eV) and carbene (blue, 283.4 eV), constraining the peak area with the ratios 4:12:10:1, respectively. This analysis is consistent with previous publications.[14] In the bottom panel, no C signal is detected before the deposition of the ligands.

F. XPS: N1s peak. IPr-NHC on Cu_xO/Cu(111) and Cu(111)

Previous publications reported the identification of adsorption configurations by meas of X-ray photoelectron spectroscopy.[15] Figure S5 shows the N1s peak for IPr-NHC on Cu_xO and Cu(111). The N1s peak from IPr-NHC on Cu_xO is slightly shifted towards higher binding energy (+0.3 eV) compared to the same molecule on Cu(111). We consider this shift to be more compatible with a different interaction with the substrate rather than a substantially different adsorption geometry. According to previous publications[1] and the DFT calculations discussed in the main text (Figure 2a-d), an upright configuration can be then assumed on both substrates.

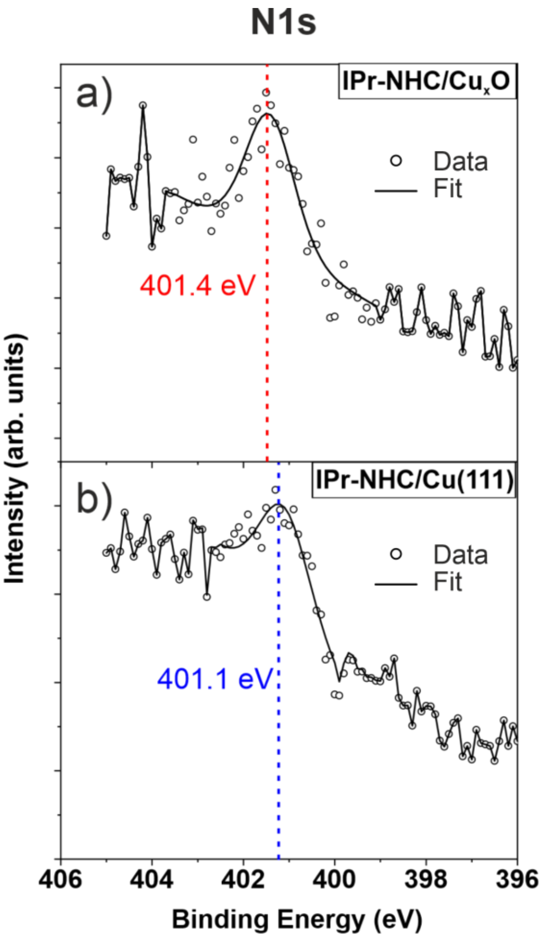

Figure S5. N1s XPS spectra for IPr-NHC molecules deposited on a) Cu_xO/Cu(111) and b) Cu(111). The dotted lines indicate the peak position: red for IPr-NHC on CuxO at 401.4 eV and blue for IPr-NHC on Cu(111) at 401.1 eV.

G. Binding modes and effect of the N-substituents

DFT calculations predict different binding modes for IPr-NHC on Cu_xO/Cu(111). A strong bond with the oxide layer is energetically favorable, where either Cu or O can act as anchor atoms, as shown in Figure 2 of the main text and in Scheme S1. As indicated in Table S1, the most favorable adsorption mode is the one at which the molecule binds to an O atom from the Cu_xO layer. In this case, the NHC-O bond length is 1.26 Å, slightly longer than the C=O bond length for a cyclic urea in gas phase (1.23 Å). In addition, the bond length between the O anchor atom and the Cu_xO layer is 2.03 Å. It is worth mentioning that the Cu-O distances in a cuprite overlayer are in the range of 1.85 – 1.90 Å. Therefore, the anchor atom keeps the IPr-NHC molecule strongly attached to the Cu_xO layer.

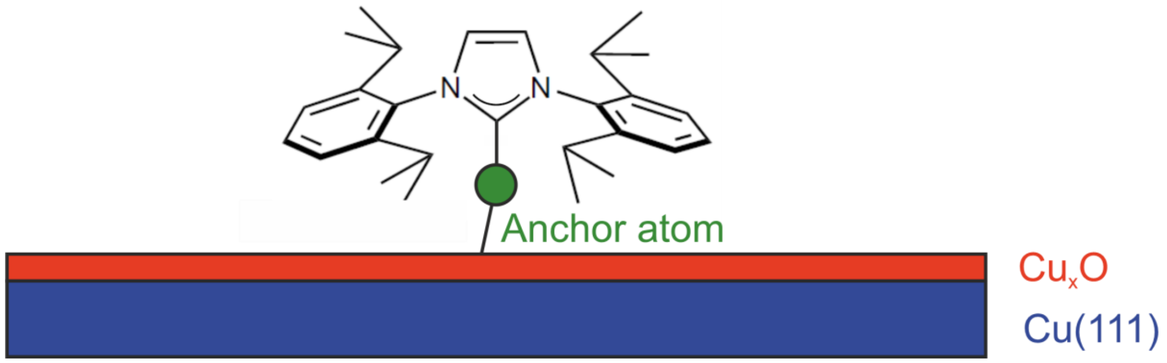

Scheme S1. Schematic illustration of the attachment of IPr-NHC on Cu_xO/Cu(111). The anchor atom refers either to Cu or to O atoms uplifted from the Cu_xO layer.

Table S1. Calculated adsorption energies and bond lengths of IPr-NHC on Cu_xO. The anchor atom refers to the atom at which the NHC binds, i.e., Cu or O.

**Adsorption Mode**	**D_e(eV)**	**NHC-Anchor Atom bond length (Å)**	**Anchor atom-Cu_xO bond length (Å)**
NHC-Cu	-3.85	1.85	1.82
NHC-O	-5.01	1.26	2.03
Physisorption	-1.96	-	-

The role of the N-substituents in the adsorption of NHCs on Cu_xO/Cu(111) was also investigated. In particular, NHC with only H as substituent groups (1*H*-imidazol-3-ium-2-ide, H-NHC) and NHC with only phenyl groups (1,3-diphenyl-1*H*-imidazol-3-ium-2-ide, IPh-NHC) were additionaly taken into account (Scheme S2). The results are collected in Table S2. Interestingly, the NHC-O binding mode for IPh-NHC (D_e = -4.90 eV) is comparable to that for IPr-NHC (D_e = -5.01 eV). On the other hand, the reduced steric hindrance for IPh-NHC allows for a more efficient binding to the larger Cu atom (D_e = -4.70 eV), displaying a comparable stability to NHC-O.

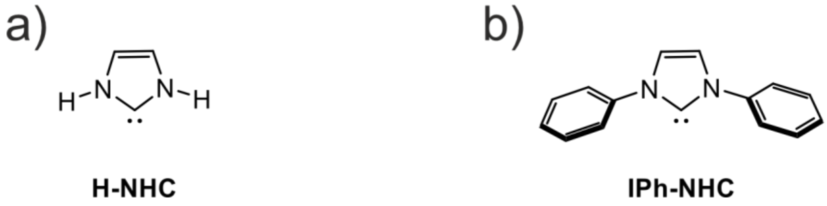

Scheme S2. NHCs considered to evaluate the effect of the N-substituents on the adsorption on Cu_xO/Cu(111). a) 1*H*-imidazol-3-ium-2-ide, H-NHC. b) 1,3-diphenyl-1*H*-imidazol-3-ium-2-ide, IPh-NHC.

Table S2. Influence of N-substituents in the adsorption of NHCs on Cu_xO/Cu(111).

**Ligand**	**Bond**	**D_e (eV)**	**Bond length (Å)**
H-NHC	NHC-Cu	-3.44	1.92
H-NHC	NHC-O	-3.62	1.25
IPh-NHC	NHC-Cu	-4.70	1.89
IPh-NHC	NHC-O	-4.90	1.25
IPr-NHC	NHC-Cu	-3.85	1.85
IPr-NHC	NHC-O	-5.01	1.26

H. Thermal Stability of IPr-NHC on Cu_xO/Cu(111)

Since the molecules arrange following the rows from the Cu_xO layer, it is reasonable to assume that they become mobile at a certain temperature between 310 K (sample temperature during deposition) and 5 K (temperature at which STM measurements are taken). This is necessary for them to find the most stable adsorption site. In order to explore this possibility, we aquired STM images of 0.05 ML IPr-NHC on Cu_xO/Cu(111) at room-temperature. Figure S6a shows a typical STM image taken under these conditions, with poor image stability and no features allowing a clear identification of the molecules. The STM image from Figure S6a was taken at 5 K, and correspdons to the same surface preparation as Figure S6a. The molecules can be clearly identified and the scanning conditions are stable. These data indicate that the IPr-NHC molecules move at room temperature due to thermal diffusion. A similar behavior was reported by the authors on Cu(111).[1]

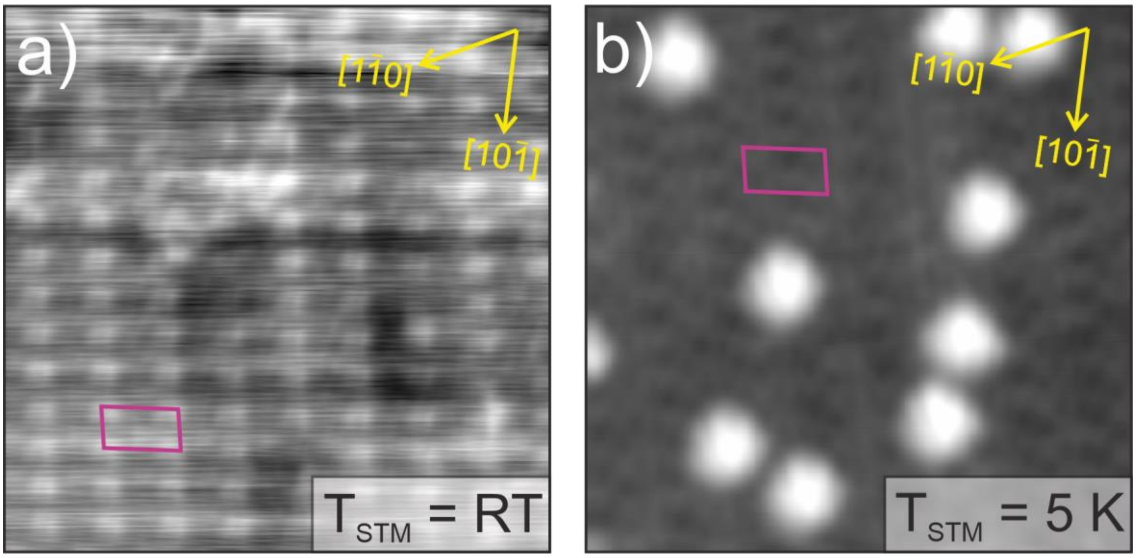

Figure S6. STM images of the Cu_xO/Cu(111) surface after 0.05 ML deposition of IPr-NHC. Images taken at a) room temperature, 13 nm x 13 nm, Vs = -1.0 V, It = 20 pA and b) 5 K, 13 nm x 13 nm, Vs = -1.0 V, It = 20 pA (Adapted from Figure 1b). Pink rectangles indicate the unit cell from the “29” Cu_xO structure.

This mobility at room temperature is still compatible with the molecules being strongly adsorbed on the surface. The STM image from Figure S7a, taken at 5 K, shows the arrangement of the molecules as described for Figure 1d, i. e., forming rows (orange lines) that match the unit cell of the Cu_xO layer. The sample was then taken out from the STM, annealed in UHV at 370 K for 1 minute and transferred back to the STM. Figure S7b shows the same molecular arrangement observed before annealing. Following the same procedure and annealing at 420 K leads to a similar arrangement. These results demonstrate that the molecules remain on the surface at temperatures as high as 420 K. As the sample cools down in the STM stage, they end up forming the same arrangement.

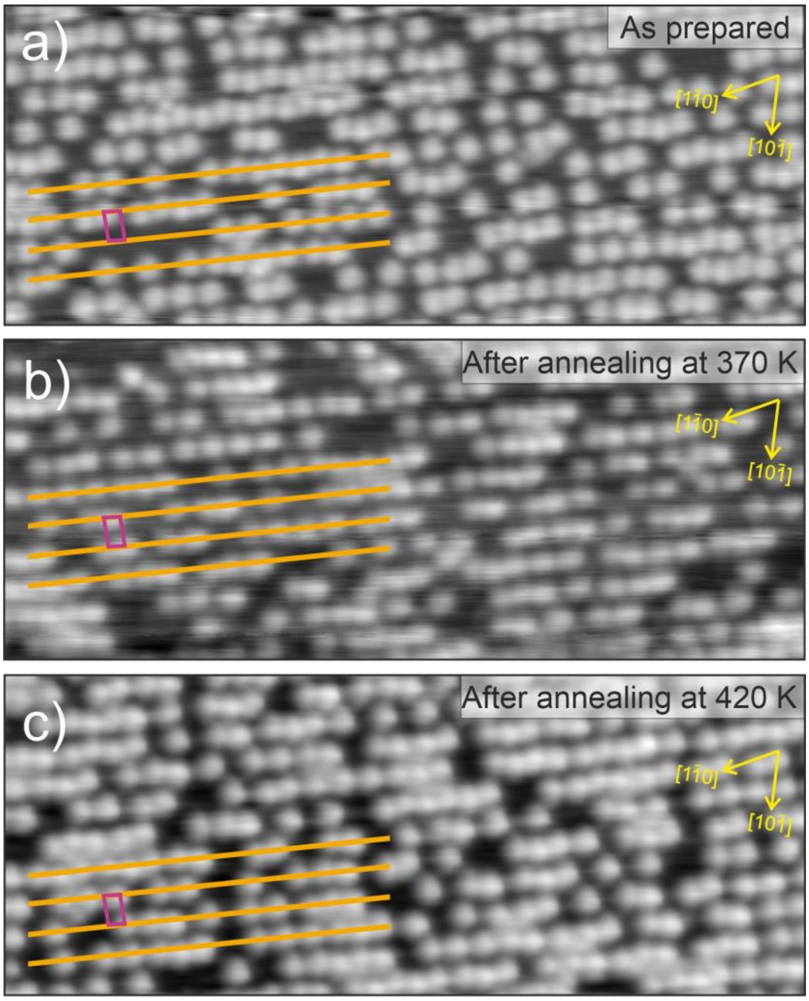

Figure S7. Thermal stability of IPr-NHC molecules deposited on Cu_xO/Cu(111). a) As prepared. 0.25 ML on Cu_xO/Cu(111). 50 nm x 20 nm, V_s = 0.5 V, I_t = 50 pA. b) After annealing at 370 K. 50 nm x 20 nm, V_s = -1.0 V, I_t = 50 pA. c) After annealing at 420 K. 50 nm x 20 nm, V_s = -1.0 V, I_t = 50 pA. STM images taken at 5 K. Pink rectangles mark the unit cell of the “29” Cu_xO structure. Orange lines indicate the direction of the stripes formed by the “29” Cu_xO lattice. The Cu(111) high symmetry directions are indicated with yellow arrows.

REFERENCES

1. Navarro, J.J.; Das, M.; Tosoni, S.; Landwehr, F.; Koy, M.; Heyde, M.; Pacchioni, G.; Glorius, F.; Roldan Cuenya, B. (2022), Growth of N-Heterocyclic Carbene Assemblies on Cu(100) and Cu(111): from Single Molecules to Magic-Number Islands. *Angew. Chem. Int. Ed.* **2022**, e202202127.

2. Kresse, G.; Furthmüller, J. Efficient iterative schemes for ab initio total-energy calculations using a plane-wave basis set. *Phys. Rev. B* **1996**, *54*, 11169-11186.

3. Blöchl, P. E. Projector augmented-wave method. *Phys. Rev. B* **1994**, *50*, 17953-17979.

4. Kresse, G.; Joubert, D. From ultrasoft pseudopotentials to the projector augmented-wave method. *Phys. Rev. B* **1999**, *59*, 1758-1775.

5. Perdew, J. P.; Burke, K.; Ernzerhof, M. Generalized Gradient Approximation Made Simple. *Phys. Rev. Lett.* **1996**, *77*, 3865-3868.

6. Grimme, S. Semiempirical GGA-type density functional constructed with a long-range dispersion correction. *J. Comput. Chem.* **2006**, *27*,1787-1799.

7. Tosoni, S.; Sauer, J. Accurate quantum chemical energies for the interaction of hydrocarbons with oxide surfaces: CH4/MgO(001). *Phys. Chem. Chem. Phys.* **2010**, *12*, 14330-14340.

8. Navarro, J. J.; Tosoni, S.; Bruce, J. P.; Chaves, L.; Heyde, M.; Pacchioni, G.; Cuenya, B. R. Structure of a Silica Thin Film on Oxidized Cu(111): Conservation of the Honeycomb Lattice and Role of the Interlayer. *J. Phys. Chem. C* **2020**, *124*, 20942-20949.

9. Yang, F.; Choi, Y.; Liu, P.; Stacchiola, D.; Hrbek, J. ; Rodriguez, J. A. Identification of 5-7 Defects in a Copper Oxide Surface. *J. Am. Chem. Soc.* **2011**, *133*, 11474-11477.

10. Matsumoto, T.; Bennett, R.; Stone, P.; Yamada, T.; Domen, K.; Bowker, M. Scanning Tunneling Microscopy Studies of Oxygen Adsorption on Cu(111) . *Surf. Sci.,* **2001**, *471*, 225-245.

11. Jensen, F.; Besenbacher, F.; Stensgaard, I. Two New Oxygen Induced Reconstructions on Cu(111). *Surf. Sci.* **1992**, *269-270*, 400-404.

12. Wiame, F.; Maurice, V.; Marcus, P. Initial Stages of Oxidation of Cu(111) *Surf. Sci.* **2007**, *601*, 1193-1204.

13. Huang, M.; Zhong, Y.; Lu, S.; Guo, Q.; Yu, Y. Antimony allotropes fabricated on oxide layer of Cu(111). *Thin Solid Films* **2021**, 138669.

14. Wang, G.; Rühling, A.; Amirjalayer, S.; Knor, M.; Ernst, J. B.; Richter, C.; Gao, H.-J.; Timmer, A.; Gao, H.-Y.; Doltsinis, N. L.; Glorius, F.; Fuchs, H. Ballbot-type motion of N-heterocyclic carbenes on gold surfaces. *Nat. Chem.* **2017**, 9, 152-156.

15. Lovat, G.; Doud, E. A.; Lu, D.; Kladnik, G.; Inkpen, M. S.; Steigerwald, M. L.; Cvetko, D.; Hybertsen, M. S.; Morgante, A.; Roy, X.; Venkataraman, L. Determination of the structure and geometry of N-heterocyclic carbenes on Au(111) using high-resolution spectroscopy. *Chem. Sci.* **2019**, 10, 930-935.
